# Registered nurses’ perspectives on barriers of cervical cancer screening in Swaziland: a qualitative study

**DOI:** 10.11604/pamj.2021.38.295.22431

**Published:** 2021-03-22

**Authors:** Siphiwesihle Sibonisiwe Mkhonta, Joyce Shirinde

**Affiliations:** 1 School of Health Systems and Public Health, Faculty of Health Sciences, University of Pretoria, Private Bag x 20, Hatfield, 0028, South Africa

**Keywords:** Cervical cancer, screening, registered nurses, perceptions, barriers, attitudes

## Abstract

**Introduction:**

cervical cancer is highly preventable and curable if diagnosed and treated early, however, it is still the leading cause of death among women. Despite efforts to increase cervical cancer screening, its uptake is still low. The purpose of the study was to investigate the perspectives of registered nurses on barriers of cervical cancer screening in Swaziland.

**Methods:**

an exploratory qualitative design was used for the study. Face to face in depth interviews were conducted with 15 registered nurses using an interview guide. The study was conducted in four clinics from both the private and public sectors, which were purposively sampled. Interviews were captured using an audio recording device, they were transcribed verbatim and then analysed using thematic analysis.

**Results:**

four themes emerged which were deterrents to cervical cancer screening. These were perceived client barriers, health care system related barriers, nurse related barriers and the nature of the procedure.

**Conclusion:**

these barriers to service provision need to be addressed by extensive health education to women as well as the cervical cancer screening programme to provide all resources required to increase the uptake of screening.

## Introduction

Cervical cancer is the second leading cancer affecting women globally and is accountable for 265 000 deaths annually, 90% of which are from low income countries [1]. Also, in 2012 there was an estimate of 445 000 new cases of cervical cancer and 80% of these were from developing countries [2]. Swaziland is ranked the country with the highest incidence of cervical cancer in the sub-Saharan region with 223 women diagnosed annually and 118 deaths [3,4]. Cervical cancer is a preventable and curable disease if diagnosed and treated early [3]. The most effective way for early detection is regular screening with a papanicolaou test (PAP smear) combined with the human papillomavirus (HPV) test. The WHO (World Health Organization) states that the high cervical cancer mortality rate could be decreased by effective screening and treatment programmes [5]. Unfortunately, developing countries still have limited access to effective screening, meaning that cervical cancer is frequently not detected until it is in the advanced stages with poor prognosis [3].

The cervical cancer screening provided in Swaziland includes visual inspection using acetic acid (VIA) and PAP smear offered in all hospitals, health centres and public health units [6]. Some clinics only offer VIA whilst some offer both PAP smear and VIA and refer suspicious results to the hospitals for further management. However, even with the simplest methods in place, health care providers still face challenges in service provision due to various factors. The factors that hinder screening have been classified into: the health care system and health care provider related [7-9]. According to two studies, inaccessibility to health care centres and the unavailability of quality services are hindrances to cervical cancer screening [8,10]. Health care centres might be available, but might not provide screening services throughout the day or even every day, thereby limiting access to screening [11,12]. Additionally, women living in hard to reach areas have to travel very long distances in order to access services thus discouraging them from screening [8,9,11]. Other stumbling blocks to screening include: long waiting times and queues in the clinic, slow turnaround time for results, poor quality smears rejected in the laboratories resulting in repeating tests, and lastly the costs of services and tests [13,14].

Several studies showed that health care providers identified the following barriers in offering cervical cancer screening: insufficient screening centre/space and equipment at their working places, financial barriers for clients in those health care centres where screening is charged [7,8,12,15-17]. Furthermore, two studies conducted in Zimbabwe showed that lack of time dedicated to counselling and educating women on cervical cancer and staff shortages served as deterrents to screening [8,9]. The health care provider related factors encompassed insufficient training or the limited number of trained staff, resulting in them being less confident and reluctant to recommend the PAP smear test for women [7,10]. Work overload was also a factor causing health care providers not to discuss with every patient about the screening [10,15]. Lastly other studies revealed the male gender of a health care provider as a barrier to women screening [8,10,18]. Other studies in Swaziland investigated the knowledge, attitudes, practices and factors of cervical cancer screening among women and men [19-21]. The health care worker´s perspective has not been investigated hence; this is the first study to explore the registered nurses´ perspectives on barriers of cervical cancer screening in Swaziland. The objective of the study was to explore the barriers to cervical cancer screening.

## Methods

**Study design and setting:** an exploratory qualitative study design was carried out among registered nurses. The epistemological framework was subjective, and knowledge was co-created by the participants and the researcher. The qualitative study design was chosen because it made possible to gain a deeper understanding of the perspectives of registered nurses on barriers of cervical cancer screening. Four clinics providing cervical cancer screening were selected from each of the four regions of Swaziland. Clinics were labelled as clinic 1,2,3 and 4. They were: clinic 1 (a rural clinic in the Manzini region), clinic 2 (a governmental clinic in the Shiselweni region), clinic 3 (a government clinic in the Lubombo region) and clinic 4 (a non-governmental organization in the Hhohho region). Registered nurses working in the department offering cervical cancer screening in the selected clinics were interviewed. Purposive sampling was undertaken in this study. Nurses working in clinics that did not offer cervical cancer screening were excluded. Therefore, the total sample size was 15 registered nurses.

**Data collection:** data were collected using individual face to face interviews to gain an in depth understanding, using an interview guide in both English and Siswati depending on participants' preference. The interview guide was developed based on existing formulated tools from previous studies on cervical cancer [15,22-24]. Additional probes and follow-up questions for clarification and elaboration on responses were used. The interview guide was pre-tested among registered nurses who did not work in the four selected clinics before the actual data collection. This helped in providing feedback on wording and improve questions that were not clear. The interviews were conducted in a private space in the clinic where there were no disturbances. An audio recording device was used for recording the interviews and notes were written while the interviews were progressing. The interviews took approximately 45 minutes to an hour for each participant. The audio transcripts of the interviews were transcribed verbatim by the researcher.

**Data analysis:** data captured were manually analysed using thematic analysis. Thematic analysis helps to identify the main themes [25,26]. Data were clustered into groups, coded and from there, the main themes and categories were developed. To reduce the total list of categories, topics that relate to each other were grouped, and lines drawn between categories to show interrelationships. The resulting categories were combined to form overarching sub-themes or themes depending on the complexity of the categories. The coding of the data according to developing codes, categories, and themes that emerged were done manually.

**Ethical consideration:** ethical clearance for the study was obtained from the Faculty of Health Sciences Research Ethics, University of Pretoria (763/2018) and the Swaziland Research Review Board (SRH 081/2019). The permission to conduct the study was obtained from authorities in the clinic´s unit of the Ministry of Health. Participants in the study received a participant information letter and gave a written consent.

## Results

There were fifteen participants in total: eleven females and four males. The study used a good mixture of years of work experience ranging from as little as six months to twenty years. Also, the participants had varying qualifications which ranged from nursing assistant, diploma in general nursing with midwifery, community health nursing with mental health and community health nursing with midwifery. Ten participants received formal training on cervical cancer screening and five had not received any training. Among those who received training only three were males (Table 1). Four themes emerged (nurse related barriers, healthcare system related barriers, client perceived barriers and the nature of the procedure) (Table 2).

**Table 1:** participant´s demographic data (n=15)

	Participant	Sex	Work experience	Qualification	Trained on screening
**Clinic 1**	P1	Male	9 years	Diploma in Nursing & Midwifery	Yes
	P2	Female	5 years	Community Health Nursing & Midwifery	Yes
	P3	Female	6 years	Diploma in Nursing & Midwifery	Yes
	P4	Female	6 months	Diploma in Nursing & Midwifery	No
**Clinic 2**	P1	Male	20 years	Diploma in Nursing & Midwifery	Yes
	P2	Female	18 years	Diploma in Nursing & Midwifery	Yes
	P3	Male	6 years	Nursing Assistant	No
**Clinic 3**	P1	Male	15 years	Diploma in Nursing & Midwifery	Yes
	P2	Female	6 years	Community Health Nursing & Midwifery	Yes
	P3	Female	8 years	Community Health Nursing & Mental Health	Yes
	P4	Female	18 years	Nursing Assistant	No
**Clinic 4**	P1	Male	15 years	Diploma in Nursing & Midwifery	Yes
	P2	Female	2 years	Medical Surgical Nursing & Midwifery	Yes
	P3	Female	3 years	Diploma in Nursing & Midwifery	Yes
	P4	Female	6 months	Medical Surgical Nursing & Midwifery	No

**Table 2:** summary of themes and categories

Theme 1	Categories
Nurse related barriers	Shortage of staff
	The gender of the nurse
	Shortage of trained nurses
	A nurse who is a resident of the community
	Lack of support from colleagues
**Theme 2**	
Healthcare system related barriers	Accessibility of services
	Availability of services
	Shortage of equipment
	Poor supervision from the programme
	The cost of the screening test
	No means of patient follow up
	Increased waiting time
	The booking system
	Busy working hours
	Poor laboratory- results system
**Theme 3**	
Perceived client barriers	Patients´ fears
	Being screened by a male nurse
	Absence of the preferred nurse
	Lack of knowledge
	The asymptomatic nature of cervical cancer
	Long walking distance to the facility
	Cultural beliefs
**Theme 4**	
The nature of the procedure	The procedure is an invasion of one´s privacy

**Nurse related barriers:** nurse related barriers mentioned by participants were as follows:

**Shortage of staff:** most of the participants mentioned the shortage of registered nurses as a barrier to screening. One participant responded: *“Its staff shortage that hinders us from offering cervical cancer screening daily”* (P3: C4).

**The gender of the nurse:** some male participants pointed out that the male gender hinders women from screening. Most women decline to be screened by male nurses because it´s perceived as an invasion of privacy. One male participant stated: *“I tell women we do the screening to your preference, but a lot of women refuse to be screened by me... they opt for the female nurses to screen them”* (P1: C3).

**Shortage of trained nurses:** participants cited the shortage of trained nurses on cervical cancer screening as a hindrance to screening. One participant stated: *“We are four, but I am the only one trained. When am on leave or off duty, cervical cancer screening is not offered in the clinic”* (P4: C3).

**A nurse who is a resident of the community:** some participants reported that being a resident of the community where the clinic is located is a barrier to cervical cancer screening: *“But the problem is I am a member of the community, so the women in the community don´t feel very comfortable being screened by me”* (P1: C3). Another participant shared: *“...the women don´t want community residents to screen them so they feel like he/she will know their personal staff”* (P3: C1).

**Lack of support from colleagues:** participants shared lack of support from the other colleagues. They shift all the responsibilities of the cervical cancer screening and follow up to the trained nurse: *“This thing ends up being your responsibility or problem, the other nurses say you are the one who was trained so do the follow up”* (P2: C3).

**Health care system related barriers:** several health care system related barriers revealed by the participants are discussed below.

**Accessibility of services:** some participants mentioned that the PAP smear screening was not available in other clinics, meaning post-menopausal women need to go elsewhere for screening because they are not eligible for VIA. A participant shared: *“We do not offer the PAP smear because we never got the smear bottles, brushes and slides for conducting it. So, we do not screen the elderly, they have to seek other healthcare centres where the PAP smear is offered”* (P2: C3).

**Availability of services:** participants stated that offering the screening services once a week was a barrier: *“We offer the service once a week. If a woman comes on a day when the service is not offered, they are turned away”* (P3: C1).

**Shortage of equipment:** participants mentioned lack of working equipment as a hindrance to service availability, hear one nurse explain *“As much as we offer the service, but we are not complete, we need equipment”* (P1: C3). The shortage of equipment ranged from: *"We don't have PAP smear bottles, brushes, and slides"* (P2: C4). Another participant explained: *“We don´t have an autoclave we always ask other facilities to sterilize the equipment this is a limitation”* (P1: C3).

**Poor supervision from the cervical cancer programme:** participants reported poor support or follow up from the cervical cancer programme on the progress of the service. One participant responded: *"With the program, I feel like they are too silent, they respond very late to my concerns on cervical cancer screening. They take too long in responding even if we report shortage of equipment"* (P2: C2).

**The cost of the screening test:** participants from the private sector mentioned that charging the service can be an obstacle to screening especially because cervical cancer is asymptomatic in the early stages. A participant explained: *"The PAP smear test cost $13.17 and VIA is $2.63, yeah charging the service can be a barrier, if women don't have the money for screening, then they can't get the service, and to some women $13.17 is a lot"* (P4: C4).

**No means for patient follow up:** some participants reported no telephone and or no money for calling patients as a hindrance to screening. They were not able to make a follow up on booked clients due to lack of airtime: *“We book adolescents for screening, but they never show up and we, we usually don´t have airtime to make a follow up and call them”* (P3: C3). Another participant also added: *"We don't have a clinic telephone to contact patients if they have to repeat PAP smear, I think it's better for those who have a telephone to do follow up care"* (P2: C2).

**Increased waiting time:** participants mentioned that increased waiting time at the facility may be an obstacle to cervical cancer screening: *“Some women end up leaving the clinic without the service due to the long queues”* (P2: C1).

**Busy working hours:** more than fifty percent of the participants stated work overload and busy working hours as a cause for offering the service once a week: *“Mondays and Tuesdays are very busy, Thursdays and Fridays some nurses are off duty, so Wednesday was a better choice for cervical cancer screening, we are very busy that´s why we screen once a week.”* (P1: C3).

**The booking system:** some participants cited booking client as a barrier, *"The booking system is not effective because one patient can be booked but never come back for screening on the booked date, hence a missed opportunity"* (P4: C1).

**The poor laboratory-results system:** all participants reported having problems with the laboratory. The problems include the long turnaround time for PAP smear results. Participants shared: *“The results take two months, three months or even more but at times they never come back”*(P4: C3). Another participant also added: *“Oh! gosh (grimacing) we have to keep following up, there is a challenge with the laboratory, we ask the driver to do a follow up or tell the patient to check with the laboratory”* (P3: C1).

**Perceived client barriers:** perceived client barriers identified by participants are as follows:

**Patients´ fears:** one of the perceived barriers mentioned by the participants was the fear of pain during the procedure: *“I think women are scared to undergo the procedure because they think inserting the speculum is painful” (P1: C3). Also: “Women always fear cancer, what if I screen and find that I have cervical cancer how do I live with that”*(P3: C3).

**Being screened by a male nurse:** some participants stated that patients may have the interest to screen but upon discovering that a male nurse is offering the screening they decline screening. One participant shared: *“to me being a male nurse, women refuse that I screen them, not rudely they say they are not comfortable.”* (P3: C1).

**Absence of the preferred nurse:** two female participants mentioned the absence of the preferred nurse as a hindrance to screening. One participant responded: *“Sometimes women prefer to be screened by a certain nurse because as people we are choosey, if the preferred nurse is absent then they won´t screen”* (P2: C2). Another added: ”So, it´s also about the preferences, given that I open my private part which nurse am I opening them to” (P2: C4).

**Lack of knowledge:** one participant stated lack of knowledge on cervical cancer and screening can hinder women from screening: *“If women in the community don´t know about cervical cancer screening, they may not come for screening even if services are free ”* (P4: C4).

**The asymptomatic nature of cervical cancer:** the absence of signs and symptoms of cervical cancer in early stages may impede women from screening, one participant reported: *“If someone is not feeling any pain she does not respond quickly, they cannot take their time and come to the clinic when there is no pain”* (P3: C3).

**Long walking distance to the facility:** participants stated that the long walking distance to the facility can deter screening: *“Our clinic is hard to reach, it´s a long distance from the homesteads to the clinic, that too can be another barrier to screening”* (P3: C3).

**Cultural beliefs:** participants mentioned culture as a barrier to screening. One participant shared *“...our culture as well, they teach us to keep your private parts private. Then you go to screen for cervical cancer where you must spread your legs for someone else, even worse if it´s a male nurse”* (P2: C2).

**The nature of the procedure:** the participants stated that the procedure compels one to expose the private parts and insert a speculum in the woman´s vagina, which is an invasion of one´s privacy making clients uncomfortable. *“The nature and posture assumed during the procedure, whether you are a female or male, someone´s nakedness is private, the procedure is an invasion of someone´s privacy”* (P3: C1).

The participant further explained: *"The person opens the legs, it is more invasive, and you are going to insert something in the vagina then she thinks otherwise, but when you both females it's difficult to think otherwise. But if you are a male and inserting something ewu. Nobody would want to be put in such an awkward situation.”*(P1: C2).

## Discussion

The main barriers to service provision were nurse related, healthcare system related, client perceived barriers and the nature of the procedure. The participants mentioned patient perceived barriers which were: lack of knowledge, patients´ fears, being screened by a male nurse, absence of preferred nurse, the asymptomatic nature of cervical cancer, long distance to clinic and cultural beliefs. Other studies have identified lack of patient knowledge as a major barrier to cervical cancer screening [13,14,16,18,27-29]. Similarly, a study conducted in Swaziland by Ngwenya showed that women were poorly aware of the signs, symptoms, and benefits of cervical cancer screening and that only 5.2% of women had screened for cervical cancer [19]. If women possess little or no knowledge on cervical cancer and screening, then they are less likely to utilize the service even if it is offered for free at the healthcare facilities. This finding is echoing the importance of intensifying health education strategies in the community on cervical cancer and screening. This study revealed that women did not accept screening when conducted by a male nurse. In contrast, acceptance was high when a female nurse was conducting the screening. Other studies also concluded that being screened by a male healthcare provider was identified as a barrier for women to screen as it made them uncomfortable [8,10]. The study showed that culturally, women are raised to keep the vagina private but with the screening, they are expected to expose it, even worse to a male nurse too. Other studies had similar results where cultural beliefs were barriers to screening [30,31]. Furthermore, studies confirmed that cultural belief is a barrier to screening because the test is done around the vagina, which is culturally seen as sacred and is only to be touched and seen by the sexual partner [18,32]. To a lesser degree, patients' fears, being asymptomatic and long distance to the clinic were also identified as patient perceived barriers to screening [8,10,30]. All the latter studies had similar results on patient perceived barriers except for the absence of a preferred nurse.

The study revealed nurse related barriers which were: a shortage of staff, the gender of the screening nurse, limited trained nurses, a nurse who is a resident of the community and lack of support from colleagues. The nurse related barriers identifed in this study are consistent with studies of other health care providers [7,16,28]. The shortage of nurses and limited trained nurses were common barriers found in two studies [12,15]. However, all studies mentioned the nurse related barriers except for lack of support from colleagues and a nurse who is a resident of the community, which were findings in this study. In addition, healthcare system related barriers were identified. The inaccessibility of a PAP smear test was a barrier to cervical cancer screening. Some health facilities did not offer the PAP smear test, and this had cost implications for post-menopausal women who then had to travel to other hospitals for the test. Additionally, a study in South Africa underlined that women who do not afford medical costs have reduced access resulting in poor health outcomes [33]. Moreover, in our study, cervical cancer screening was not offered every day thus posing an access barrier for women. Parallel studies also noted that the unavailability of and inaccessibility to cervical cancer screening services were the main barriers to screening [7,8]. Charging the cervical cancer test was also a barrier to screening in this study. This finding was also verified by a study in Iran which observed that charging the cervical cancer test may pose as a barrier to screening [34].

The unavailability of a telephone or money for calling patients at the facility was another healthcare system related barrier to patient follow up. Participants were not able to call patients and remind them of screening appointments. Other studies affirmed that having no telephone was a barrier to patient follow up and screening [11,15]. There must be adequate resources for any organization to function effectively [35]. However, inadequate equipment, including a broken sterilizer, was a barrier to cervical cancer screening found in two studies [15,16]. Likewise, for this study, equipment shortage such as autoclave, PAP smear brushes, and slides were barriers to screening. Failure to provide equipment makes it difficult for employees to carry out their work in an easy, non-obstructive way [36]. The absence of equipment leads to a halt in cervical cancer screening, resulting in reduced productivity. Our study found that increased waiting time, busy working hours, poor laboratory-result system, the booking system and poor supervision from the cervical cancer screening programme can impede screening. Consistent studies also confirmed that long waiting hours, limited working hours, poor laboratory and booking system and poor supervision of cervical cancer programme were possible barriers to screening [7,8,34]. Furthermore, a study conducted in Iran found that the healthcare system authorities need to pay more attention to the programme by continuous funding. This can ensure working equipment and human resources are available to increase screening uptake [34]. Also, the nature of the screening procedure was a barrier to screening because it was an invasion of women's privacy. Likewise, some studies confirmed that the screening procedure made women uncomfortable as it invaded their privacy [8,11,16].

Limitations: registered nurses from referral hospitals were excluded, and this is an important limitation as they are more likely to have different perceptions on cervical cancer screening. The study is based on self-reported information, this is a limitation because of recall and social desirability bias, however, the interview guide was piloted. A qualitative research design was used; therefore, the findings cannot be generalized. The strength of this study was the detailed description of the context since it used in depth interviews.

## Conclusion

Barriers of screening were healthcare system related, nurse related, the nature of the procedure and client perceived. Therefore, it is imperative to address these barriers by increased health education, as well as the cervical cancer programme to provide all resources required to increase the screening uptake.

### What is known about this topic

- A cross sectional study was conducted on the knowledge, attitudes and practices on cervical cancer and screening among men and women in Swaziland;
- Although cervical cancer screening services are available in the public health units, clinics and hospitals, the screening uptake is still low;
- Swaziland is ranked the country with the highest incidence of cervical cancer in the sub-Saharan region, with 223 women diagnosed with cervical cancer and 118 deaths per annum.

### What this study adds

- To our knowledge, this is the first study to qualitatively explore the registered nurses´ perspectives on barriers of cervical cancer screening in Swaziland;
- The shortage of trained nurses and equipment disturbs nurses in their determination to deliver quality cervical cancer screening services. These barriers play a major role in the low screening coverage;
- The findings of this study will influence the development of effective strategies and or policies to increase the screening uptake.
